# Coexistence of Two Spin Frustration Pathways in the Quantum Spin Liquid Ca_10_Cr_7_O_28_

**DOI:** 10.1021/acs.inorgchem.2c01831

**Published:** 2022-10-03

**Authors:** Dhoha R. Alshalawi, José M. Alonso, Angel R. Landa-Cánovas, Patricia de la Presa

**Affiliations:** † Institute of Applied Magnetism, UCM-ADFI-CSIC, A6 22,500 km, Las Rozas28230, Spain; ‡ Institute of Material Science of Madrid, CSIC, Madrid28049, Spain; § Department of Materials Physics, Complutense University of Madrid, Madrid28040, Spain

## Abstract

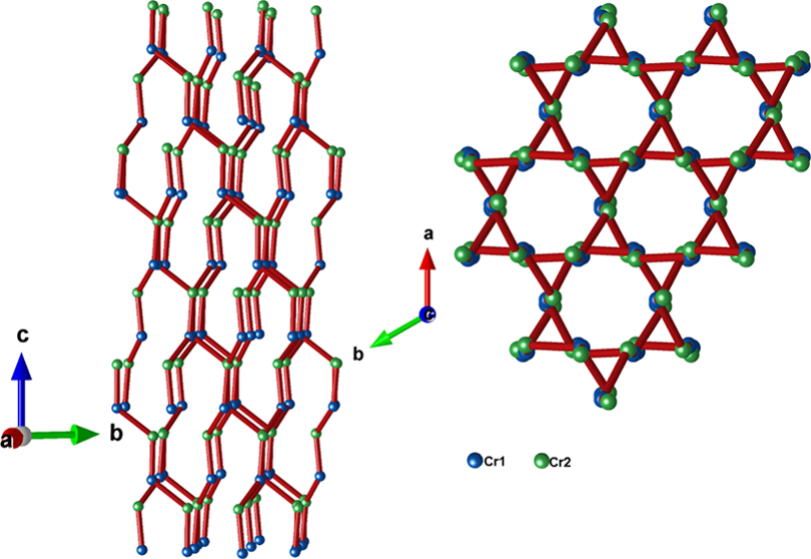

Kagome antiferromagnetic lattices are of high interest because the geometric frustration is expected to give rise to highly degenerated ground states that may host exotic properties such as quantum spin liquid (QSL). Ca_10_Cr_7_O_28_ has been reported to display all the features expected for a QSL. At present, most of the literature reports on samples synthesized with starting materials ratio CaO/Cr_2_O_3_ 3:1, which leads to a material with small amounts of CaCrO_4_ and CaO as secondary phases; this impurity excess affects not only the magnetic properties but also the structural ones. In this work, samples with starting material ratios CaO/Cr_2_O_3_ 3:1, 2.9:1, 2.85:1, and 2.8:1 have been synthesized and studied by X-ray diffraction with Rietveld refinements, selected area electron diffraction measurements, high-resolution transmission electron microscopy (HRTEM), low-temperature magnetometry, and magnetic calorimetry. This result shows that a highly pure Ca_10_Cr_7_O_28_ phase is obtained for a CaO/Cr_2_O_3_ ratio of 2.85:1 instead of the 3:1 usually reported; the incorrect stoichiometric ratio leads to a larger distortion of the corner-sharing triangular arrangement of magnetic ions Cr^+5^ with *S* = 1/2 in the Kagome lattice. In addition, our study reveals that there exists another frustration pathway which is an asymmetric zigzag spin ladder along the directions [211], [12–1], and [1–1–1], in which the Cr–Cr distances are shorter than in the Kagome layers.

## Abstract 2

This work represents the endeavor to ensure the correct stoichiometric composition of the quantum spin liquid material Ca_10_Cr_7_O_28_. The synthesis and characterization addressed several nonstoichiometric samples, including impurities’ influence on the crystal structure and properties of Kagome and zigzag magnetic interaction. The characterization aspects of the compound are based on the X-ray diffraction data and Rietveld refinement. Further characterization could help us understand the nature of quantum material and aid in the additional development of quantum theories and technology.

## 1 Introduction

For years, one of the areas of condensed matter physics that arouses the most interest has been that of frustrated magnetic materials. Frustrated magnetic materials are those in which the spins interact through exchange interactions competing with each other and cannot be satisfied simultaneously, causing a great degeneration in the fundamental state of the system. For example, a triangular array of magnetic ions with antiferromagnetic (AFM) exchange leads to frustration since it is not possible to satisfy simultaneously the interactions of the lattice ions.^1^

Quantum spin liquids (QSL) are the paradigm of magnetic frustrations, and their properties are currently undergoing many theoretical and experimental investigations. Even though the theoretical frame has been well-established in the ‘70s,^2^ the experimental results on QSL are still scarce. There are many types of suggested materials considered to have QSL properties such as mixed magnetic valence ions, coherent spin dynamics in the ground state, and complete absence of long-range order.^1,2^ The interest of these compounds lies in the rich physics and exotic properties of QSL, such as long-range entanglement and fractional quantum excitations, which are believed to hold great potential for quantum communication and computation.

Several compounds have been proposed as QSL candidates or potential candidates,^3^ including κ-(BEDT-TTF)_2_Cu_2_(CN)_3_,^4,5^ herbertsmithite ZnCu_3_(OH)_6_C_l2_,^6,7^ ruthenium-based QSL as alpha-RuCl_3_ and Na_4_Ir_3_O_8_,^8,9^ and Ca_10_Cr_7_O_28_.^10,11^ From all these compounds, Ca_10_Cr_7_O_28_ is one of the most interesting because, unlike the others QSLs, it is reported to present a distorted Kagome structure with dominant ferromagnetic interactions.

Surprisingly, the study of the bibliography about the synthesis of Ca_10_Cr_7_O_28_ has shown that, to our knowledge, there is no work that shows a synthesis with starting materials relation CaO/Cr_2_O_3_ 2.85:1 that leads to the correct ratio Ca/Cr 10:7, but the starting materials ratio used has been 3:1, which leads to compounds with CaCrO_4_ and/or CaO excess.^10−17^

We think that the origin of this incorrect starting material ratio lies in the intention to synthesize Ca_3_(CrO_4_)_2_, a material that should have been isostructural with the spin dimmers Sr_3_(CrO_4_)_2_ and Ba_3_(CrO_4_)_2_.^18−20^ However, to our knowledge, Ca_3_(CrO_4_)_2_ could not be synthesized and instead Ca_10_Cr_7_O_28_ with Ca excess has been produced.

Mirtič et al.^16^ investigated the synthesis of Ca_3_Cr_2_O_8_ single crystals by varying the starting material ratio (but always the CaO/Cr_2_O_3_ ratio is higher than 3), obtaining apparently Ca_3_(CrO_4_)_2_ with remnant untransformed CaCrO_4_. Later, different attempts have been done by varying starting materials ratio and sintering temperatures and times in order to obtain the phase Ca_3_Cr_2_O_8_, resulting finally in a large variety of Ca–Cr–O phases as Ca_3_(CrO_4_)_2_, Ca_10_Cr_6_O_25_, and Ca_5_Cr_3_O_12_,^21−23^ all of them showing different segregated secondary phases and Cr valences (3+, 4+, 5+ and 6+).

The first report on Ca_10_Cr_7_O_28_ is from Gyepesova and Langer.^14^ The authors proposed that the sought phase Ca_3_(CrO_4_)_2_ has in fact the chemical formula Ca_10_(Cr^5+^O_4_)_6_(Cr^6+^O_4_) with structural space group *R*3*c*, and assigned to Cr cations two oxidation states, with only one Cr^6+^ and six Cr^5+^. Similar to the previous work, the authors used a CaO/Cr_2_O_3_ ratio of 3:1 and observed secondary segregated phases.

Recently, intensive studies of the composition Ca_10_Cr_7_O_28_ have been done in order to identify the two different chromium valences as magnetic Cr^5+^ and nonmagnetic Cr^6+^, where Cr^5+^ has *S* = 1/2 and with a structure containing Kagome bilayers.^10−13,15,17,24^ The theoretical and experimental results show that this compound displays all the features expected for QSL.^10−13,15,17^ Balz et al.^11^ synthesized single crystal and powder samples from high purity starting materials with a CaO/Cr_2_O_3_ ratio of 3:1.^11^ However, neutron and X-ray synchrotron characterization show that the samples contained 13% of the CaO secondary phase.^13,24^ Single crystal of Ca_10_Cr_7_O_28_ was grown to study the field dependence of the electronic and thermal properties at ultralow temperature, including different structural models for the system.^25^ Further studies of physical properties and theoretical simulations^26,27^ have been performed based on the previous results of Balz et al.^11^

These previous results show that Ca–Cr–O compounds synthesized with CaO/Cr_2_O_3_ ratio ≥3 always lead to the desired Ca_10_Cr_7_O_28_ phase with the mixed valence Cr^5+^ and Cr^6+^ but also to secondary segregated phases, normally of CaO and/or some oxide of Ca–Cr. The impurity phases do affect the correct structural characterization of the samples since the presence of the impurity can lead to twins in crystals, local distortion of the structure, or excess Ca in the main structure and can indeed affect the magnetic properties. As shown by Uchida et al. in relation to the Ba_3_Mn_2_O_8_,^28,29^ the presence of impurities can give rise to important variations in the values of the dominant interactions in this type of material with in-plane exchange *J*_0_. As the understanding of the QSL is intimately bounded to the structure, it is relevant to obtain pure high crystalline Ca_10_Cr_7_O_28_ for the proper characterization of the structure and a better understanding of the magnetic properties in the ground state of quantum spin liquid.

This work is focused on two main aspects of Ca_10_Cr_7_O_28_: (i) the synthesis and characterization of a high-quality crystalline sample of Ca_10_Cr_7_O_28_ without secondary phases, for this aim, starting materials ratios 2.8 ≤ CaO/Cr_2_O_3_ ≤ 3 have been explored for the first time. (ii) To discern, by means of Rietveld refinement and HRTEM of the impurity-free sample, between the double cell proposed by Balz et al.^13^ and the model proposed by Gepesova.^14^ Additionally, magnetic and calorimetric characterizations of the impurity-free sample are reported.

## 2 Results and Discussion

### 2.1 Compositional Analysis

**Figure 1:**
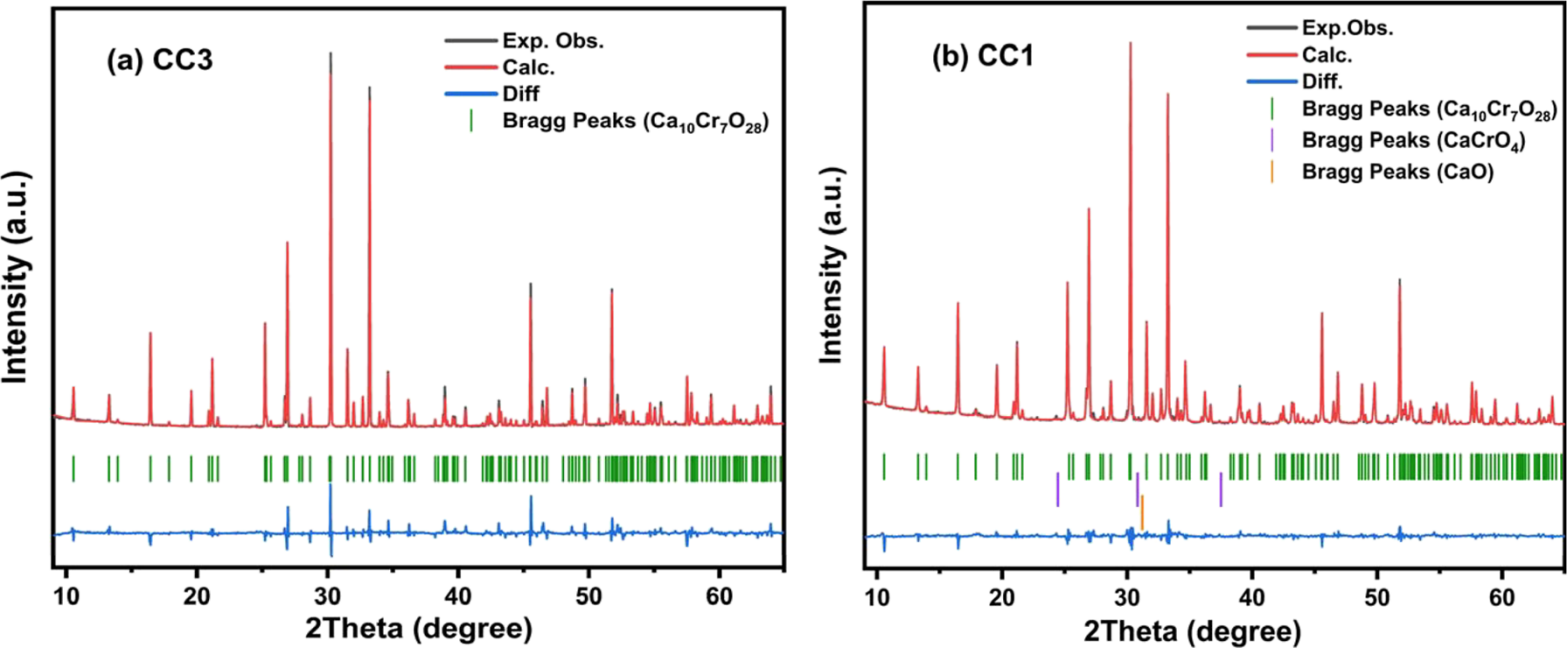
XRD patterns and Rietveld fit for (a) CC3 and (b) CC1 samples: the experimentally observed peaks (black lines), profile calculated peaks (red lines), and the difference of Rietveld (blue lines) and Bragg peaks (green lines) for Ca_10_Cr_7_O_28_, (purple line) CaCrO_4_, and (orange line) CaO.

From the analysis of X-ray diffraction (XRD) of four samples with different CaCO_3_/Cr_2_O_3_ ratios CC1 (3.0:1), CC2 (2.90:1), CC3 (2.85:1), and CC4 (2.80:1), the sample CC3 with the ratio 2.85:1 was the only one showing a single-phase without impurities of the starting materials oxides CaO, Cr_2_O_3_, or segregated phases, as can be seen in Figure 1a. On the other hand, the samples with a ratio of 3.00:1 (CC1, used in the majority of the works^10−17^) (Figure 1b) and 2.90:1(CC2) show four extra maxima at 2θ ≈ 24.6, 31, 35.5, and 37°. The maximum at 2θ = 32° fits pretty well with the 100% intensity of CaO (2θ = 31.2°). On the other hand, the other three maxima correspond to CaCrO_4_, which is in agreement with the electron probe microanalysis (EPMA) that shows the presence of a secondary phase with Ca/Cr ≈ 1. Additionally, the sample CC4 (2.80:1) shows only contamination of CaCrO_4_. Consequently, only the synthesis with the starting materials ratio CaCO_3_/Cr_2_O_3_ of 2.85:1 corresponds to the stoichiometric Ca_10_Cr_7_O_28_ without impurities.

**Figure 2:**
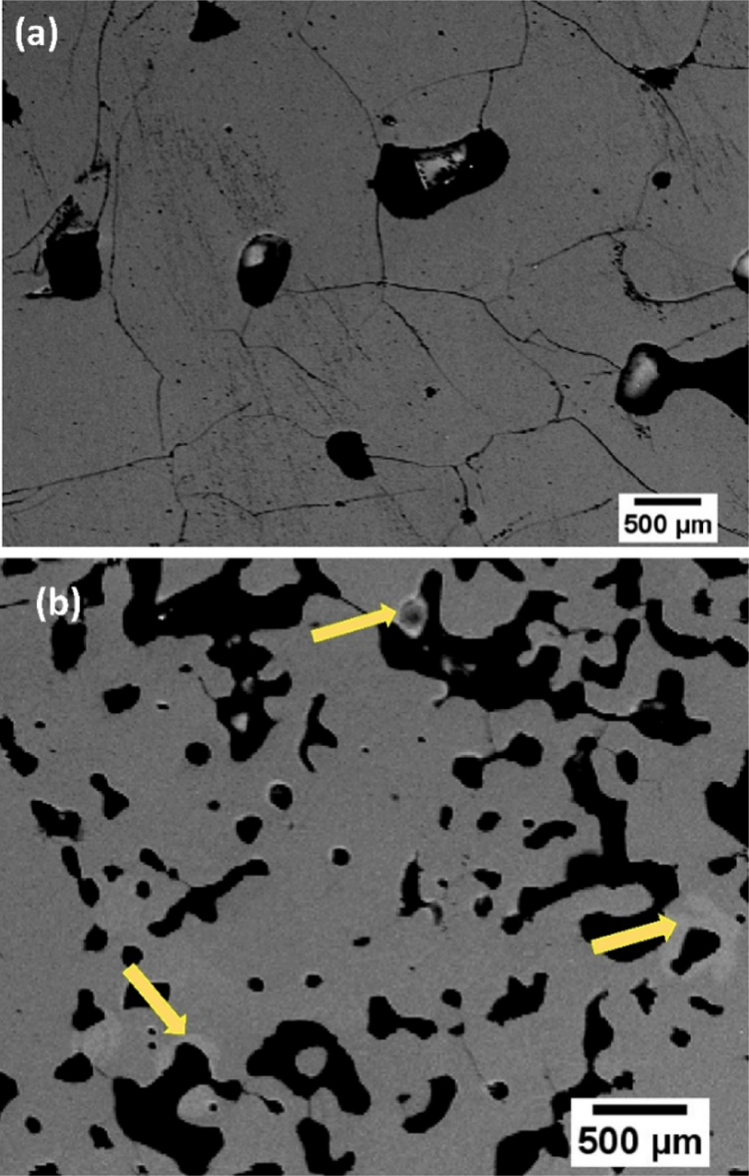
SEM micrographs of (a) CC3 and (b) CC2 by the EPMA method. The composition of the zone pointed out by the arrows is given in Table 1.

The compositional analysis has been performed by EPMA together with scanning electron microscopy (SEM); Figure 2 shows SEM micrographs of samples CC3 and CC2. As can be seen in Figure 2a, sample CC3 does not show contrast variations or areas with different tones or colors that usually appear in regions of different compositions, indicating a very homogeneous composition. Only some pores of various sizes are observed in black because the ceramic is not dense. Some slightly scratched areas resulted from the sample polishing for the observation in the microscope. Finally, the irregular lines correspond to the grain boundaries. As can be seen from Table 1, the composition is almost constant from point to point. The margin of error of this technique is around 10%, which gives a final mean composition of Ca_10(1)_Cr_6.7(7)_O_28_, that is, the composition is the stoichiometric one within the experimental errors.

**Table 1:**
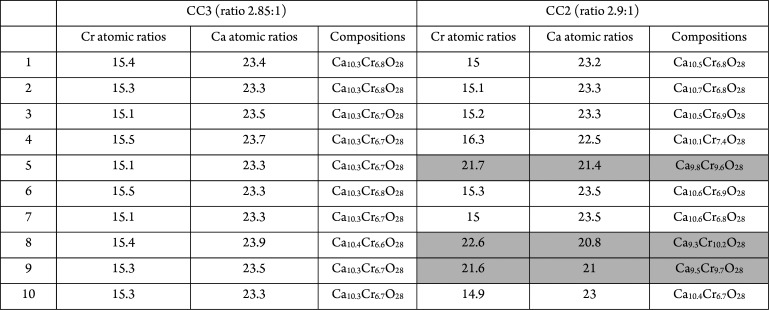
Compositional Data of Samples CC3 y CC2^a^

a The compositions marked in gray correspond to the areas marked with arrows in Figure 2b. The experimental errors are around 10%.

The results of sample CC3 differ considerably from those obtained for the other samples, for example, CC2 in Figure 2b. The arrows point out regions with different contrasts and, as can be seen in (Table 1), these regions present a Ca/Cr ≫ ratio of 1 that, according to that observed by XRD, corresponds to a CaCrO_4_.

As the sample CC3 does not present segregation of secondary phases or starting products, an inductively coupled plasma optical emission spectrometry (ICP-OES) analysis was performed to determine the accuracy of the sample’s cationic composition, and the value obtained was Ca_10.0(1)_Cr_7.0(1)_O_28_, the same as the nominal composition.

The thermogravimetric results of the CC3 sample show that mass is almost constant up to 450 °C (see Supporting Information, Figure S1). Then, the sample lost 10.2% of the initial weight with increasing temperature. The XRD pattern shows that the final product is composed of CaO and Cr_2_O_3_; therefore, considering the thermogravimetric analysis, the reduction of Ca_10_Cr_7_O_28_ occurs according to the following _reaction_
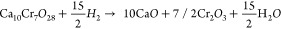


Which is associated with a 10% weight loss and confirms the oxygen content and the cationic composition as well. This result also shows that, under a reducing atmosphere, the composition is chemically stable up to 500 °C.

From the set of results obtained so far, that is, from XRD, SEM, EPMA, ICP-OES, and thermogravimetry, we can conclude that the pure phase Ca_10_Cr_7_O_28_ without impurities or compositional defects can only be obtained from the stoichiometric starting material ratio of CaO/Cr_2_O_3_ ≡ 2.85:1.

### 2.2 Structural Characterization

#### 2.2.1 Transmission Electron Diffraction

A series of electron diffraction patterns in the selected area electron diffraction (SAED) mode (selected area electron diffraction) has been obtained from different crystals of the sample CC3. The composition of each crystal was checked by X-ray energy-dispersive spectroscopy (XEDS) analysis, confirming the nominal composition.

**Figure 3:**
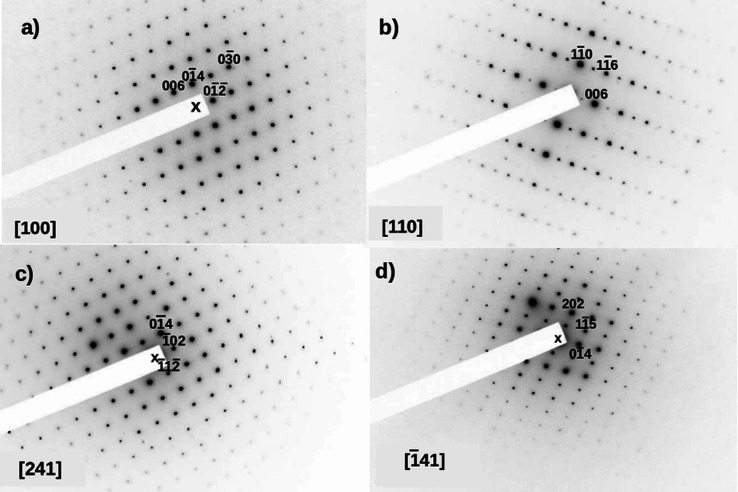
SAED patterns of Ca_10_Cr_7_O_28_ (CC3) along (a) [100], (b) [110], (c) [241], and (d) [−141] zone axes. X denotes the position of the incident beam. Notice in (a) absence of the 00*l*: *l* = 3*n* reflections due to the c-glide and how they appear weak in (b), see text for explanation. In the four SAED patterns, all the reflections fulfill the R centering condition: *hkl*: −*h* + *k* + *l* = 3*n*.

Figure 3 shows a series of SAED patterns along different zone axes, that is, different orientations of the crystals. All the observed SAED patterns present well-defined diffraction maxima that can be indexed according to the unit cell previously refined by Gyepesova and Langer.^14^ No evidence has been found of the new supercell described by Balz et al.^13^ with *a*′ = 2*a*, *b*′ = 2*b*, and *c*′ = *c*, where *a*, *b*, and *c* refer to Gyepesova’s unit cell.^14^

As shown in Figure 3a,c,d, no superlattice maxima can be seen along the [0–14]* reciprocal direction, in contrast to the reported results described by Balz et al.^13^ It is worth noting that, in the SAED pattern of Figure 3b, weak intensities can be observed for the *00l* reflections with *00l* = 3*n* and *n* odd. These reflections are forbidden for the *R*3*c* space group, but they appear in electron diffraction patterns due to multiple diffraction effects, that is, due to the dynamic nature of electron diffraction. In Figure 3a, the 00*l* reflections with *00l* = 3*n* are absent in contrast to the SAED pattern of Figure 3b. This is due to the *c*-glide plane perpendicular to the [100] zone axis, which does not allow any reciprocal path to deviate scattered intensity through allowed lattice vectors to the above-referred forbidden reflections, that is, there are no multiple diffraction phenomena transmitting intensity to the forbidden reflections. Therefore, in the SAED pattern oriented along the [110]* direction, there is no *c*-glide plane perpendicular to that direction that impedes the transmission of scattered intensity through the allowed reflections, which finally add intensity to forbidden reflections such as the 003 or the 113 reflections that can be observed clearly in Figure 3b.

Figure 3c,d show other two different crystal orientations along low symmetry directions and, again, neither sign of defects nor of the superlattice described by Balz et al. are observed.

#### 2.2.2 X-Ray Diffraction

The electron diffraction has allowed discarding the existence of a double cell with *a*′ = 2*a*, *b*′ = 2*b*, and *c*′ = *c*; therefore, the Rietveld refinements of the high-resolution diffraction pattern of all the samples at room temperature were achieved using, as initial parameters, the values given in the ref (14) (COD-Inorg # 248644 (2020.03), getting a trigonal unit cell with an *R*3*c* spatial group, and *a*,*b* = 10.76845(4) Å and *c* = 38.0848(2) Å for sample CC3. Table 2 shows the lattice cell parameter and volumen for samples CC1, CC2, CC3 and CC4 obtained by Rietveld refinements.

**Table 2:** Rietveld Refinement for Samples CC1, CC2, CC3, and CC4^a^

	lattice cell parameters			
sample	*a*, *b* (Å)	*c* (Å)	unit cell volume (Å^3^)	χ^2^
CC1 (3:1)	10.76954(7)	38.1032(4)	3827.23(5)	3.63
CC2 (2.9:1)	10.76723(8)	38.0784(5)	3823.11(7)	3.98
CC3 (2.85:1)	10.7685(4)	38.0848(2)	3824.63(3)	4.53
CC4 (2.8:1)	10.7740(2)	38.0944(10)	3829.51(8)	4.84

a The numbers in parenthesis are the standard deviations.

Tables S1 in Supporting Information gives the atomic positions, occupation factors, and the isotropic displacement parameter B of the samples CC3(2.85:1), CC1 (3:1), CC2 (2.9:1), and CC4 (2.8:1). The bond lengths of Cr cations are given in Table S2 for all the samples.

**Figure 4:**
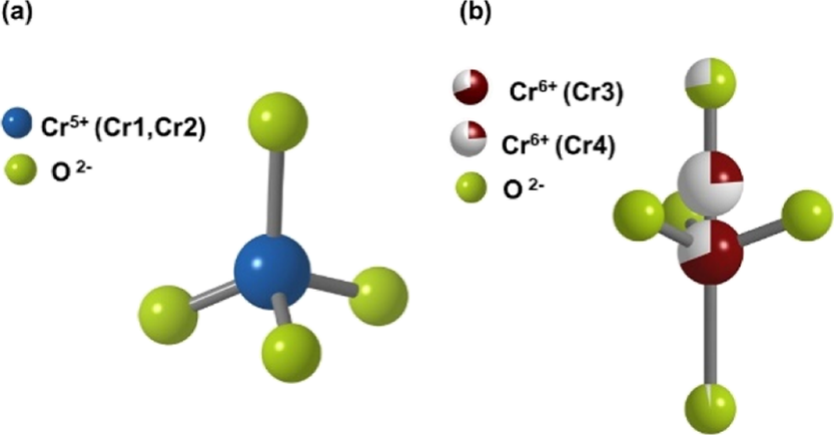
(a) Cr^5+^ in tetrahedral coordination with O^2–^. (b) Disordered tetrahedron Cr^6+^O^2–^.

From the Rietveld refinements (Table S1), it is possible to observe that the atomic positions and occupation factors are quite similar to that previously reported by other authors.^12−14,30^ As shown in Figure 4a, the Cr cations are tetrahedrally coordinated by the O^2–^, whereas the Ca^2+^ ions are bounded to six, seven, or eight O^2–^, depending on their atomic positions. Only one of the seven Cr cations is a nonmagnetic Cr^6+^, whereas the rest are magnetic Cr^5+^ with *S* = 1/2. The Cr^6+^O_4_ tetrahedron is located on a ternary axis in two possible positions whose partial occupations add up to approximately one. The disordered tetrahedron Cr^6+^O_4_ shares three O9 atoms related by symmetry in a plane perpendicular to the *c*-axis, as shown in Figure 4b.

**Figure 5:**
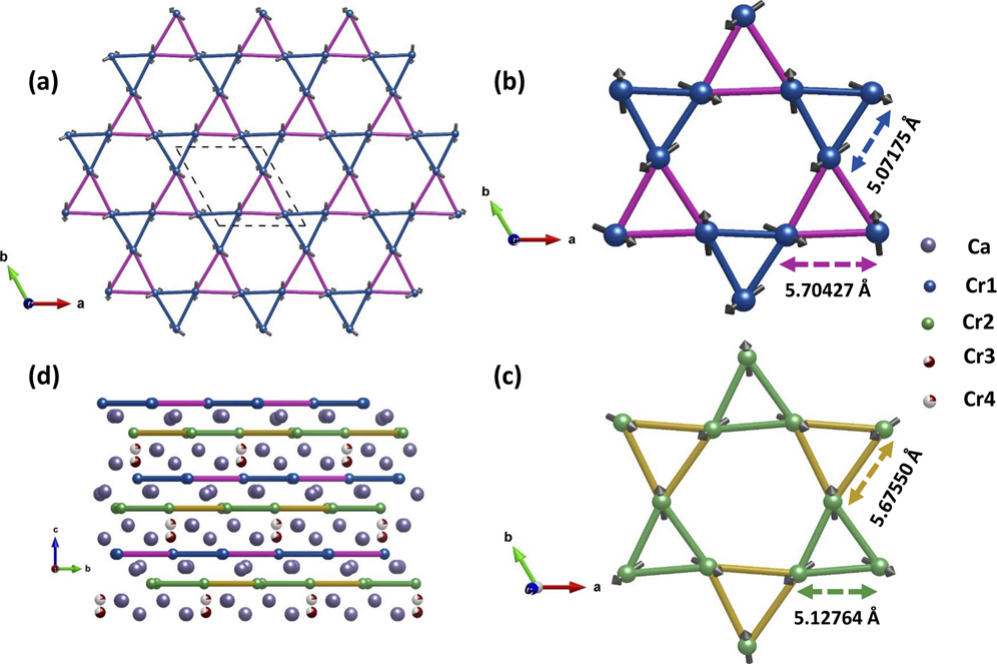
(a) One distorted Kagome layer formed by Cr1 atoms in the *a*,*b* plane projection, the dotted line represents a single unit cell. (b) Structure of a distorted Kagome layer formed the Cr1 atoms with atomic distances. (c) Structure of a distorted Kagome layer formed by the Cr2 atoms with atomic distances. (d) Kagome layers observed along the *c* axis in the range (2 2 1/2) of the half unit cell.

As described by Balodhi et al.,^30^ the structure is constituted by layers of Cr^5+^O_4_ alternating along the *c*-axis. Inside the layer, the tetrahedral orders in such a way that Cr^5+^ cations form a distorted Kagome lattice (Figure 5a–c), and Ca cations are in the middle of these layers, as shown in Figure 5d.

The Kagome layers are made up of two types of distorted triangles (see Figure 5b,c). The triangles alternate within the layers and from one layer to another, as shown in Figure 5d.

Table 3 shows the distances between the Cr atoms both inside and between the Kagome layers.

**Table 3:** Cr–Cr Distances (Å) at 300 and 14 K^a^

*d* (Å)	300 K	14 K	Δ	type
*d*_0_(Cr2–Cr1)	3.85007(2)	3.83777(8)	0.0123	interlayer
*d*_1_(Cr2–Cr1)	4.04504(2)	4.14864(4)	–0.1036	interlayer
*d*_2_′(Cr1–Cr1)	5.07175(3)	4.92083(4)	0.15092	in-plane
*d*_2_″(Cr2–Cr2)	5.12764(2)	5.08872(3)	0.03892	in-plane
*d*_3_′(Cr2–Cr2)	5.67550(3)	5.68390(4)	–0.0084	In-plane
*d*_3_″(Cr1–Cr1)	5.70427(2)	5.84320(2)	–0.13893	In-plane

a Δ indicates the difference between the distances measured at 300 and 14 K. The numbers in parentheses are the standard deviations.

The Cr1–Cr1 and Cr2–Cr2 are the distances between the Cr atoms inside the Kagome layers (intradistances), while the Cr1–Cr2 are the distances between the Cr atoms of two contiguous Kagome layers (interdistances). As can be seen, the interdistances are notably shorter than the intradistances (between 20 and 33% smaller).

**Figure 6:**
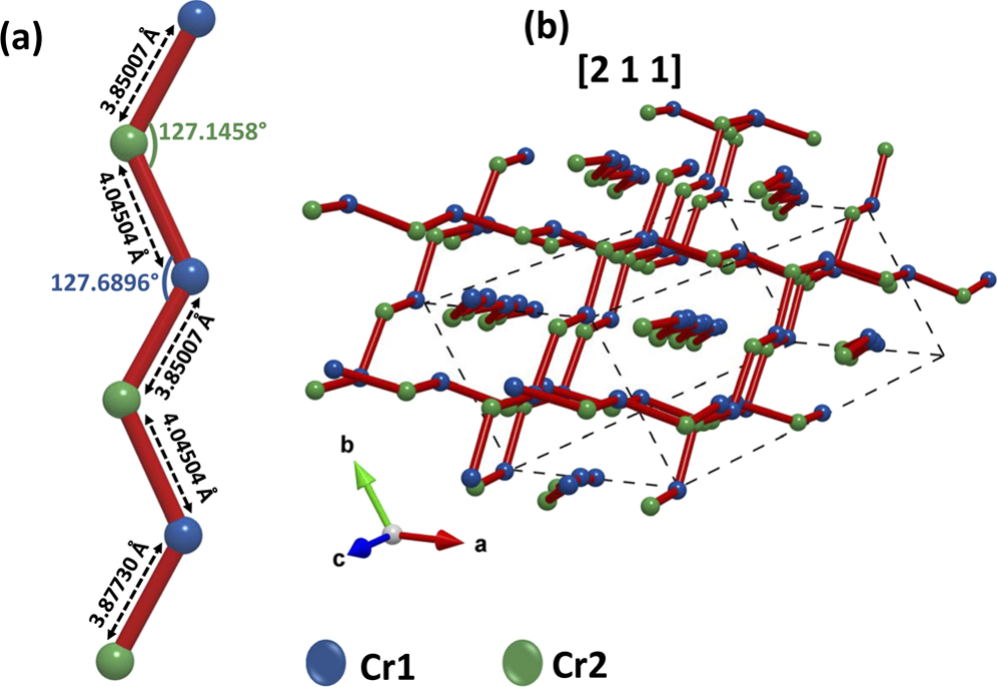
(a) Cr1 and Cr2 zigzag chain with distances and angles. (b) Perspective of the zigzag chains along the [211] axis.

The Cr1 and Cr2 link path gives rise to zigzag chains along directions [211], [12–1], and [1–1–1] of the unit cell, as shown in Figure 6. These chains are isolated from each other and surrounded by atoms of Ca^2+^ ions. It is worth noting that the zigzag angle is 127°, close to the 120° in the Kagome layers.

It should be noted that the zigzag chains with antiferromagnetic interactions are the source of strong frustration and can give rise to a one-dimensional spin liquid state as has been shown by different authors.^31−34^ The zigzag chains model has been described as a system with one-dimensional *S* = 1/2 Heisenberg antiferromagnetic interacting through the zigzag coupling of *J*1 and *J*2 between nearest–neighbor interactions in a regime of *J*2 ≫ *J*1.^35,36^

Since the interdistances of Cr1–Cr2 along the zigzag ladder [*d*_0_(Cr2–Cr1) = 3.83777(8) Å at 14 K] are much shorter than the intradistances Cr–Cr inside the Kagome layers (from 4.92083 to 5.84320 Å at 14 K), it is possible to assume that the exchange interactions along the zigzag ladder are AFM and stronger than inside the Kagome layers. Besides, these chains have their main component along the c-axis, and this could be in agreement with the results by Balz,^24^ who shows that, in single crystals, the interactions are AFM along the *c*-axis. Therefore, it is possible that there exist more than one spin frustration pathway, as recently reported by Pohle et al.^37^

**Figure 7:**
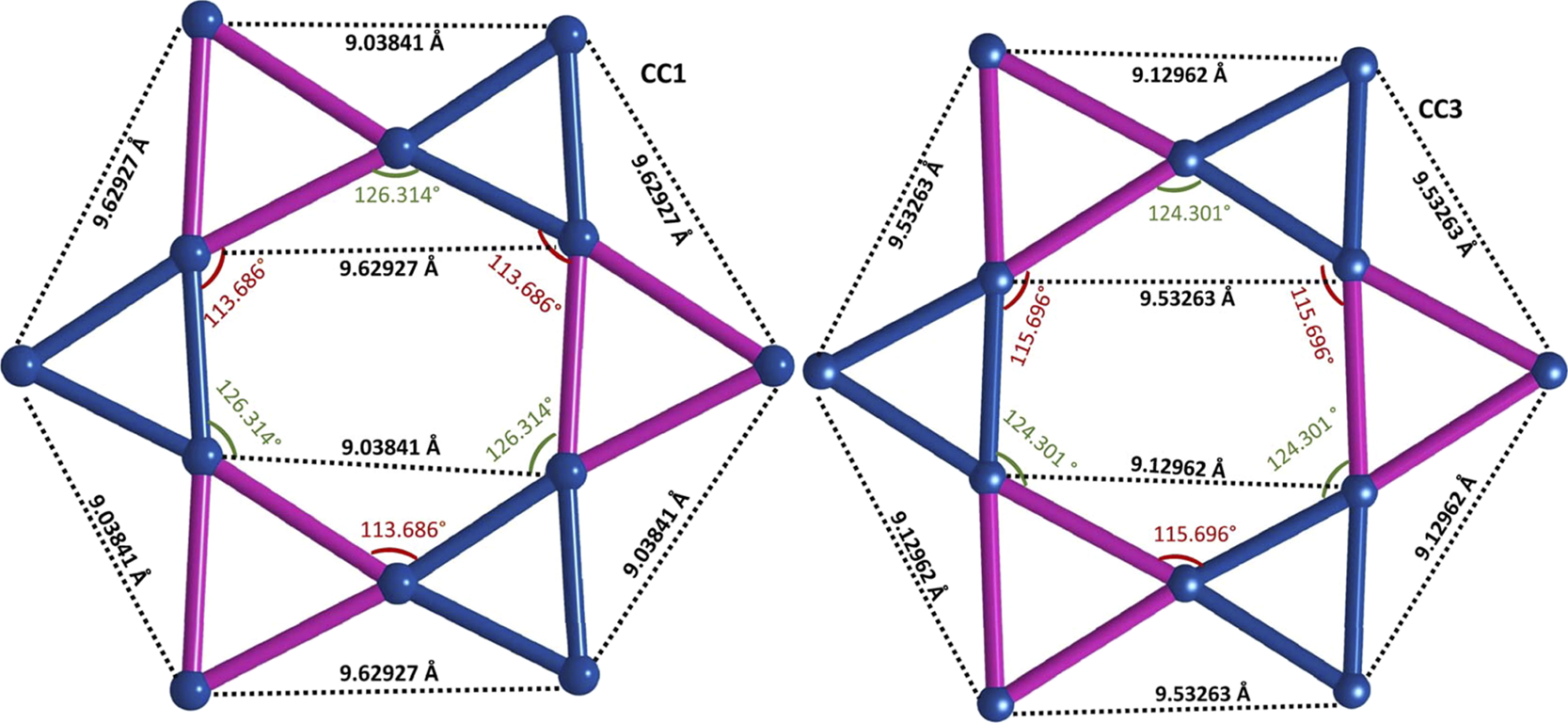
Distorted triangular structure formed by Cr1 atoms for samples CC1 (nonstoichiometric, left) and sample CC3 (stoichiometric, right). As can be seen, the differences in distances and angles are smaller in sample CC3 than in CC1.

From the results of the refinements of the sample with a starting ratio of 3:1 (CC1, the most common sample in the literature), and with a starting ratio of 2.85:1 (CC3, this work), it is possible to determine the differences between the stoichiometric and nonstoichiometric samples. For example, Figure 7 shows the distorted Kagome layers formed by Cr1 positions for both samples. As can be inferred from the figure, the distortion of the stoichiometric sample becomes smoother in the plane *a*–*b*, that is, the differences in distances and angles are smaller for sample CC3 than for sample CC1. The smoothening of the distortion could have tremendous effects on the exchange interactions between spins, and this study is underway.

#### 2.2.3 Thermo-Diffraction Analysis

The thermal behavior of the structure has been characterized by XRD from 14 to 673 K since, as shown in the thermogravimetry, the sample is chemically stable up to 723 K. The cell parameters and volume obtained by the refinements of high and low-temperature diffraction patterns in the whole temperature range are shown in Figure S2 for sample CC3. The thermal variation of the structural parameters is almost constant up to 100 K (Δ*V* = 0.08%) and then increases smoothly with increasing temperature with no evidence of phase transition. The total variation of the volume is about 2% in the whole temperature range. However, the volume is barely reduced by 0.83% between 300 and 15 K. These values are slightly smaller than those obtained using neutron TOF powder diffraction,^13,24^ which has used the starting material ratio of 3:1 (similar to our CC1 sample). Therefore, the fact that the thermal volume variation of our sample (CC3) is smaller than that reported by neutron TOF could be a consequence of the smoothening of the distorted Kagome layers in the pure sample.

A Rietveld refinement has been performed at 14 K, and the Cr–Cr distances are shown in Table 3. Comparing them with those obtained at 300 K, it is observed that some distances decrease as the temperature decreases but others increase, that is, the thermal dependence of the cell atomic distances is anisotropic. Therefore, the volume exhibits a small temperature variation but the cell parameters vary more significantly, although asymmetrically. It is also observed that the variations of Cr–Cr intradistances in Kagome layers are more significant than the interdistances between layers, which suggests that there should be a greater variation in the values of *J* within the Kagome layers than between layers.

#### 2.2.4 High-Resolution Microscopy Transmission Electron Microscopy

HRTEM has been used in order to study the presence of extended defects that are very common in materials with such big unit cells. It is important to remark that these Ca_10_Cr_7_O_28_ phases have never been explored by HRTEM, to the best of our knowledge.

**Figure 8:**
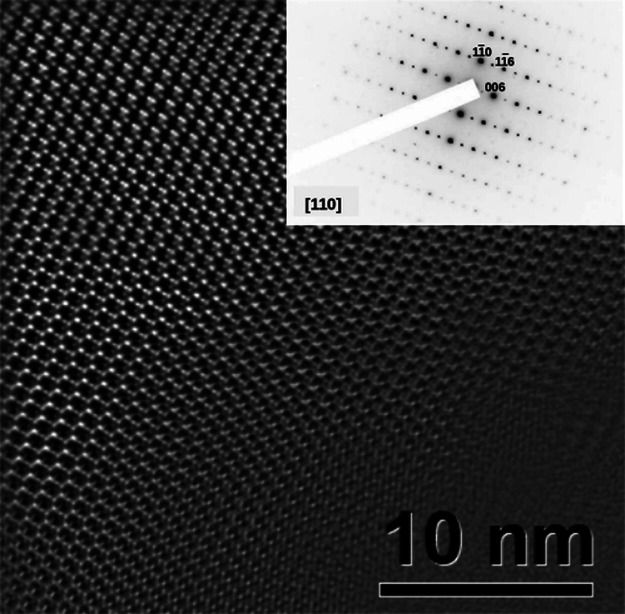
HRTEM image of a Ca_10_Cr_7_O_28_ (CC3) crystal orientated along the [100] zone axis. The inset shows a SAED image along the [100] zone axis.

Figure 8 shows an HRTEM image of a Ca_10_Cr_7_O_28_ crystal along the [100] direction, it is worth noting the perfect crystallinity and the early amorphization toward the downright corner. The image has been processed to eliminate noise.

The Ca_10_Cr_7_O_28_ crystals are sensitive to electron beam irradiation, becoming amorphous at the thin edges after a few minutes of exposition. In spite of the quick experimental acquisition of HRTEM images, we could not avoid radiation damage to the crystals, and no images could be obtained without an important noise contribution. Therefore, image-processing techniques have been used to eliminate noise. They consist in obtaining the fast Fourier transform of the image (FFT) and filtering out most of the noise using digital masks to reconstruct the image from just the structural maxima by an inverse fast Fourier transform (IFFT).

**Figure 9:**
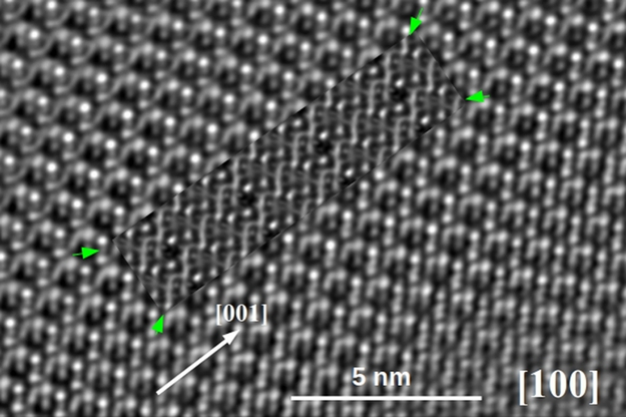
HRTEM image of Ca_10_Cr_7_O_28_ orientated along the [100] direction. Inserted, marked with green arrows, is an HRTEM image simulation for a Ca_10_Cr_7_O_28_ crystal with a thickness of 150 Å at a defocus of −600 Å.

Figure 9 shows a magnified processed HRTEM image along the same [100] zone axis with higher structural detail. The original image and its FFT are shown in Figure S3. In the inset of Figure 9, there is an image simulation for a crystal generated with the atomic parameters from the Rietveld refinement data, with 125 Å thickness at −600 Å defocus, calculated by multislice methods to take account of the dynamical effects of electron scattering. For that purpose, a whole series of images have been calculated with the parameters of the transmission electron microscope and varying both thickness and defocus, the two unknown variables. The results are exposed in Figure S4, where the images have been calculated for four thicknesses ranging from 50 to 125 Å and the objective lens defocus from −100 to −800 Å. The calculated image that fits better with the experimental image of Figure 9 is the one corresponding to a thickness of 125 Å and an objective lens defocus of −600 Å, and it has been inserted on top of the experimental image of Figure 9.

It is worth noting the very high crystallinity of the Ca_10_Cr_7_O_28_ phase, which does not show in the HRTEM micrograph any kind of lattice defect or imperfection. Besides, the calculated image fits very well with the experimental one.

### 2.3 Heat Capacity

**Figure 10:**
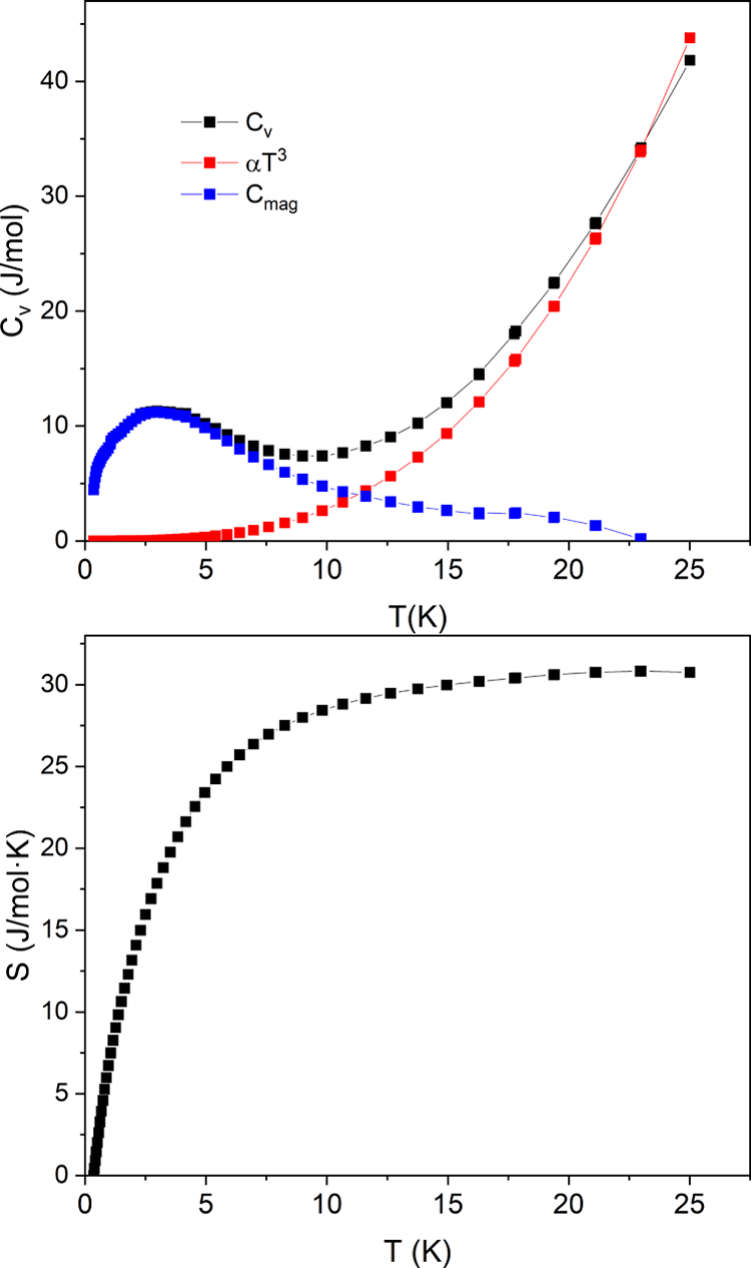
Above: specific heat capacity with the total heat capacity *C*_v_, the Debye term α*T*^3^, and the difference *C*_mag_. Below: the magnetic entropy *S*.

The specific heat capacity *C*_v_ provides information on the physical properties of a material, especially about the presence of any type of phase transition. Besides, it is related to the entropy *S* by the second law of thermodynamics providing information about the entropy evolution as a function of temperature. Figure 10 shows the *C*_v_ and *S* for the stoichiometric sample CC3. A broad maximum in *C*_v_ around *T* ≈ 3 K can be seen, which could be due to the onset of short-range magnetic correlations. Above 20 K, the contribution to *C*_v_ is purely phononic and can be subtracted from the curve by considering the Debye term α*T*.^3^ The pure magnetic contribution to *C*_mag_ is presented by the blue squares in Figure 10.

The magnetic entropy from 0.35 to 22 K sums up to:
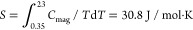


The spin entropy per mole of 6 Cr^5+^ is given by 6*R* ln(2*S* + 1) = 34.5 J/mol K; therefore, 10% of the entropy is lacking and could be associated with a long magnetic ordering below 350 mK; however, Balz *et al.* have not observed any magnetic order down to 19 mK.^11^

### 2.4 Magnetic Properties

Figure S5 shows the magnetization *versus* applied field at 2 K for the samples CC1 and CC3; the magnetic curves are unsaturated at 5 T and present neither coercivity nor remanence, as is expected for a QSL. As can be seen, sample CC1 (3:1) has 19.1 emu/g at 5 T, 12% smaller than the magnetization of CC3 (2.85:1), 21.7 emu/g. The decrease in the magnetization of CC1 cannot be explained by normalizing with the mass of the segregated phases since they are smaller than 1%. Therefore, in the sample CC1, the Ca atoms are not only segregated in impurity phases but also probably introduced in the structure, affecting the magnetic interactions. This is in agreement with that observed by Uchida *et al.* in the Ba_3_Mn_2_O_8_.^28,29^

**Figure 11:**
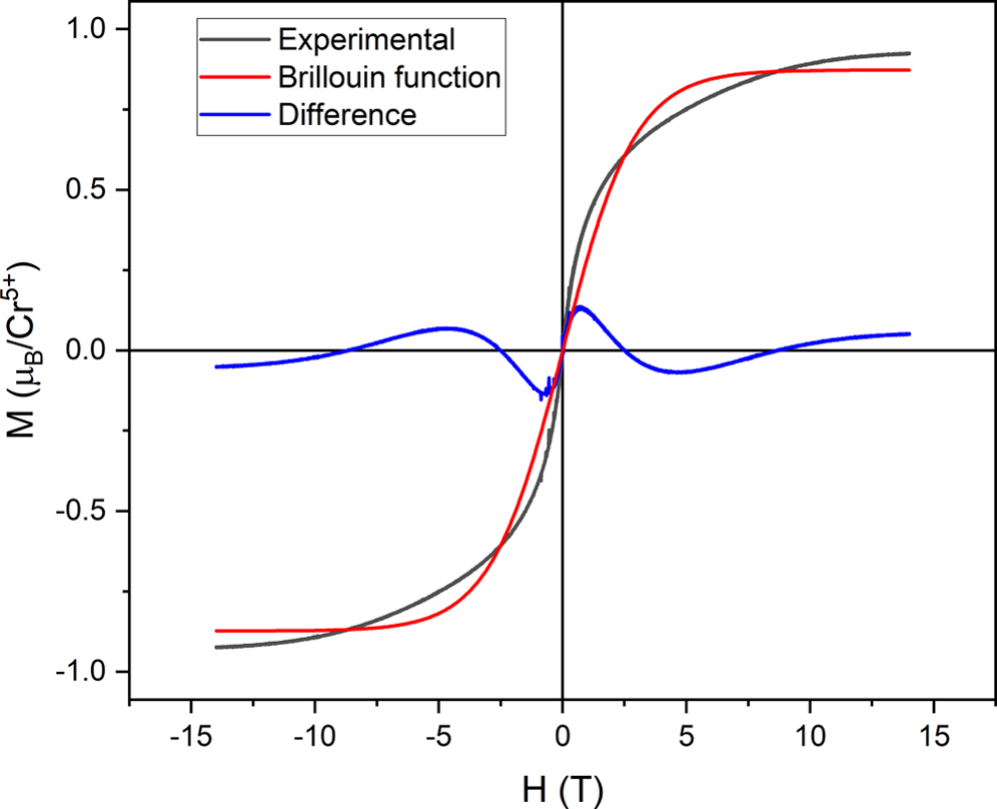
Hysteresis curves at 1.8 K and 14 T; the black line is the experimental curve, the red one is the Brillouin function, and the blue one is the difference.

Figure 11 shows the hysteresis curves of CC3 at 1.8 K and 14 T; at this temperature and field, the system is almost saturated. Since the Cr^5+^ ions have spin *S* = 1/2, each of them contributes to magnetic moment with *g*_S_μ_B_*S* = 1μ_B_ (with *g*_*s*_ = 2), whereas the experimental value is 0.92 μ_B_, probably because the temperature is relatively high and the ground state has not yet been reached. The onset of ferro-, ferri- or antiferromagnetic states can be discarded given the lacking of remanence and coercivity in the hysteresis loop. A Brillouin function has been fitted to the hysteresis curves and, as can be seen in Figure 11, there is a huge difference between the Brillouin function and the experimental results, discarding also a paramagnetic state. Therefore, the QSL state is the most probable state, as it is known, the QSL is a lattice of coherently fluctuating magnetic moments, and no long correlation magnetic order is observed at the ground state, despite the magnetic moments being arranged in an antiferromagnetic network.^1,2^

**Figure 12:**
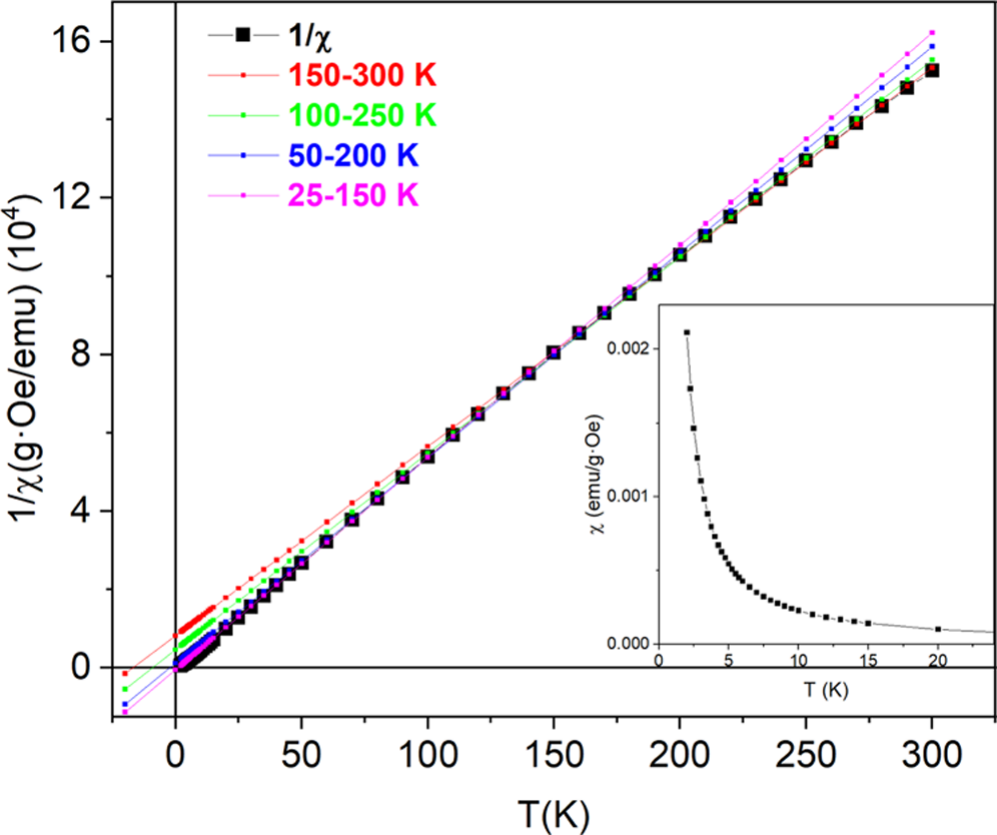
Thermal dependence of the inverse of the magnetic susceptibility and fits at different temperature ranges. The inset shows the susceptibility from 2 to 24 K measured at 500 Oe.

The thermal dependence of the susceptibility χ and its inverse 1/χ calculated per gram of Cr^5+^ is shown in Figure 12. As can be seen, χ as well as 1/χ show a smooth variation with the temperature, without evidence of magnetic transitions. As it is known, the paramagnetic susceptibility follows a Curie–Weiss law χ = *C*/(*T* – θ_CW_), where *C* is related to the effective magnetic moment μ_eff_, and the sign of θ_CW_ is related to the exchange interaction functions *J*. A positive θ_CW_ indicates predominantly ferromagnetic interactions, whereas a negative value indicates an AFM exchange. In a paramagnetic regime without changes in the *J*’s, 1/χ must be a straight line; however, a slight curvature is observed in Figure 12, indicating a change in the spin magnetic arrangement probably related to the thermal contraction of the unit cell. In order to obtain θ_CW_, a linear fit on 1/χ was performed at different temperature ranges: 25–150, 50–200, 100–250, and 150–300 K.

The θ_CW_ obtained from these fittings changes from negative to positive depending on the temperature range of the fitting, as shown in Figure 12. Thus, for a temperature interval between 150 and 300 K, θ_CW_ is negative (θ_CW_ = −16 K), indicating that the interactions between Cr^5+^ are predominantly antiferromagnetic. In contrast, for the interval 25–150 K, the value of θ_CW_ is small but positive (θ_CW_ = 2 K) and, therefore, the predominant interactions are ferromagnetic. This behavior indicates a change in the exchange interactions between Cr^5+^ cations in Ca_10_Cr_7_O_28_ that goes from being predominantly antiferromagnetic at high temperatures (θ_CW_ < 0) to predominantly ferromagnetic at low temperatures (θ_CW_ > 0). A similar effect has been observed by Balz^24^ by measuring single crystals with the field parallel or perpendicular to the *c*-axis.

The slope, 1/*C*, of the linear fit of 1/χ in the temperature range 150 to 15 K gives *C* = 140 emu K/g Oe; the experimental value of μ_eff_ can be obtained with the expression μ_eff_ = (3*CAk*_B_/*N*)^1/2^,^38^ where *N* is the Avogadro number, *A* it the atomic weight, and *k*_B_ is the Boltzmann constant. The obtained experimental value μ_eff_ = 1.64μ_B_ is close to the value of the “spin-only” contribution: μ_eff_ = g[*S*(*S* + 1)]^1/2^μ_B_ = 1.73μ_B_.

**Figure 13:**
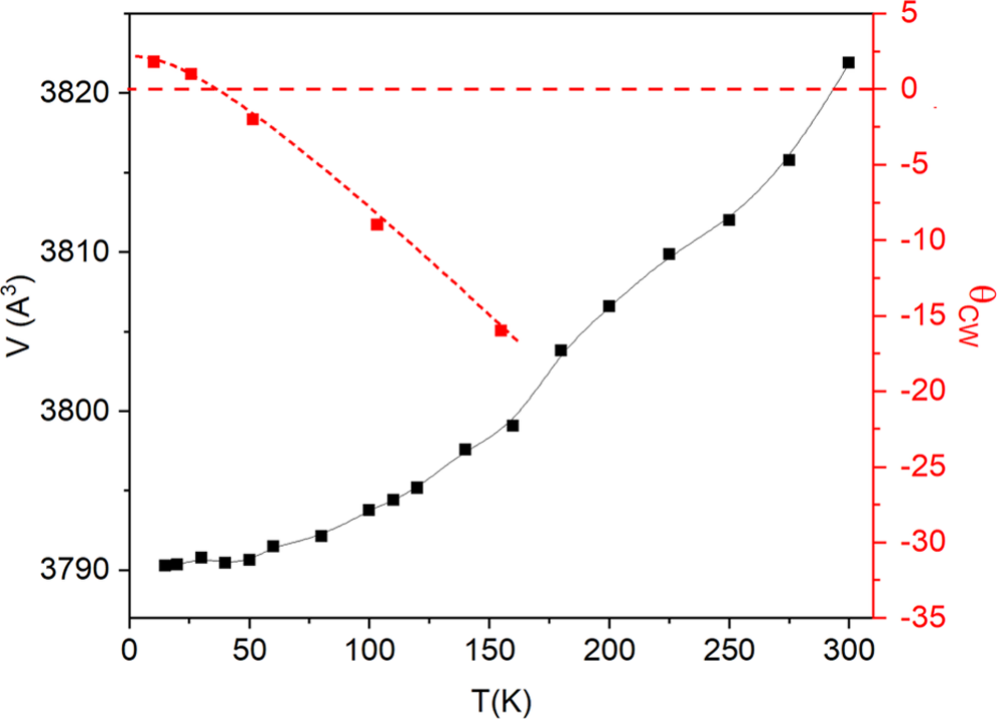
Temperature dependence of the unit cell volume (left axis) and θ_CW_ (right axis) of Ca_10_Cr_7_O_28_.

When *q*_CW_ obtained for each temperature range is plotted against the minimum temperature of such interval (see Figure 13), it is observed that the sign of *q*_CW_ changes at *T* ≈ 40 K. This change is related to the contraction of the unit cell at low temperature. As commented previously, the cell volume decreases with decreasing temperature, but it stabilizes for temperatures smaller than 50 K. In addition, it has been observed that the Cr–Cr distances vary in an anisotropic way with the temperature (see Table 3). The correlation between both variables (*q*_CW_ and *V*) is shown in Figure 13, suggesting that the change of sign in the exchange interactions is probably due to the cell expansion and the anisotropic variation of the Cr–Cr distances.

## 3 Conclusions

A revision of the bibliography of the starting materials ratio for the synthesis of Ca_10_Cr_7_O_28_ has been done and it is concluded that most of the samples in the literature have been synthesized with a CaO/Cr_2_O_3_ ratio of 3:1, which leads to samples with CaO impurity and others Ca–Cr oxides. In this work, we propose that the correct starting material ratio is 2.85:1, which results in stoichiometric Ca_10_Cr_7_O_28_ without impurities. The composition and the phase purity are also confirmed by EPMA, ICP-OES, XEDS, and thermogravimetric analysis (TGA) characterizations. The ICP-OES analysis results confirm that the mean composition is Ca_10.0(1)_Cr_7.0(1)_O_28_, which fits the nominal one. The TGA shows the chemical stability of the composition Ca_10_Cr_7_O_28_ up to 723 K.

The crystalline cell group of the stoichiometric material (ratio 2.85:1) is *R*3*c*, with lattice parameters ***a*** = ***b*** = 10.76845(4) Å, ***c*** = 38.0848(2) Å, α = β = 90°, and γ = 120°, in good agreement with that reported by Gyepesova and Langer.^14^ The refinements of the XRD pattern of the stoichiometric and nonstoichiometric samples show that the stoichiometric samples have the smallest cell parameters and volume. Besides, the refinements show that the distortion in Kagome layers formed by the magnetic Cr^5+^ is smoother in the stoichiometric sample than in the others. The smoothening in the distortion has consequences on the exchange interactions. Additionally, it is shown that the shortest distances Cr1–Cr2 are for those Cr^5+^ cations between the Kagome layers, forming zigzag ladders, whereas the Cr1–Cr1 or Cr2–Cr2 distances inside the Kagome layers are larger. These results require neutron characterization of the impurity-free sample in order to determine the dominant exchange interactions in the structure.

High-resolution XRD thermo-diffraction patterns have been obtained from 14 to 673 K. The cell parameters and thermal dependence of the stoichiometric sample were obtained by Rietveld refinements. It is observed that the cell parameters as well as the unit cell volume smoothly decrease with decreasing temperatures; however, the atomic distances have an anisotropic variation with the temperature.

Both electrons diffraction SAED and HRTEM of pure Ca_10_Cr_7_O_28_ show that, in the absence of secondary phases, there are not any diffuse intensity lines and superlattice maxima as described in ref (13); indeed, the transmission electron microscopy (TEM) results confirm the crystalline model proposed by Gyepesova and Langer.^14^ It is worth noting that, despite the size of the cell and the amount of atoms inside, the structure does not show any defect, and the cations are completely ordered.

The magnetization in the high field shows that the decrease of the magnetization for the nonstoichiometric samples is more pronounced than that corresponding to the segregated phase, indicating that excess Ca cations in the structure could affect the exchange interactions of the Cr^5+^ cations. The hysteresis curve measured at 2 K and 14 T shows neither coercivity nor remanence, and a paramagnetic Brillouin function does not fit the hysteresis curves. All these results lead to the conclusion that the strong frustrated behavior could be a spin liquid state. On the other side, the inverse of susceptibility, 1/χ, is slightly curved, indicating changes in the exchange interactions, which can be correlated to the thermal cell expansion: at the smallest cell volume, θ_CW_ > 0 suggests a ferromagnetic arrangement of the spin moments, whereas θ_CW_ becomes negative at temperatures higher than 50 K, which corresponds to an antiferromagnetic arrangement of the spin moments.

Analogous to that observed by other authors, the specific heat behaviors make Ca_10_Cr_7_O_28_ a candidate to be a spin liquid. This behavior is interpreted in terms of frustration within the Kagome layers, as they are triangular networks. However, there is another possible origin of the magnetic frustration that could take place in the zigzag Cr1–Cr2 chains that present the shortest Cr–Cr distances of the structure observed along the directions [211], [12–1], and [1–1–1]. If the exchange between the Cr atoms of these chains is AFM, a strong frustration appears and is the origin of one-dimensional spin liquids: it is called the two-legged zigzag ladder.^31−34^ Further studies are required to disentangle this question.

## 4 Experimental Details

### 4.1 Sample Synthesis

Polycrystalline samples of calcium chromates were synthesized using the starting materials CaCO_3_ (PanReac, 99.9%) and Cr_2_O_3_ (Alfa Aesar, 99.9%). The starting materials were mixed with different molar ratios CaCO_3_/Cr_2_O_3_ (CC); the sample names are given according to decreasing molar ratios: CC1 (3:1), CC2 (2.9:1), CC3 (2.85:1), and CC4 (2.8:1). The samples were prepared in air and synthesized by the ceramic method. The corresponding CaCO_3_ and Cr_2_O_3_ powders were homogenized, milled in an agate mill, and sintered at 1100 °C in air for seven days. The samples were finally quenched at room temperature.

### 4.2 Analytical Characterization

The chemical analysis was performed by inductively coupled plasma optical emission spectrometry (ICP-OES) using the reference IT-1 = 04345J0 3701 with a range of uncertainty ±0.1. The samples were digested in duplicate with an HNO_3_/HCl mixture in a closed Teflon reactor and placed in an oven for a couple of days at a temperature of 110 °C. These measurements were performed at the Center of Geological Techniques at the Complutense University of Madrid, Spain.

Chemical cation compositional analysis was determined using an electron probe micro-analyzer (EPMA) attached to a JEOL JXA-8900 microscope, around 20 areas of 1–5 μm were analyzed. The measurement was performed at the National Center for Electron Microscopy (ICTS) at the Complutense University of Madrid, Spain.

The oxygen content was determined by TGA on a Cahn D-200 electro balance by reducing samples under H/He (0.2/0.3 atm.) from room temperature up to 900 °C. Since the final product of the reduction process, determined by X-ray diffraction, was always the same mixture of CaO and Cr_2_O_3_ oxides, the oxygen content was determined from the weight difference between the starting material and the final products.

### 4.3 Structural Characterization

The structural phase of the samples was characterized using the high-resolution Panalytical X’Pert PRO MPD diffractometer with a Cu Kα radiation tube (λ = 1.5406 Å) working at 45 kV and 40 mA, primary beam monochromator, and fast X’Celerator detector. The samples were mixed with acetone and placed in the Si holder. The patterns were obtained at room temperature with a 2θ range from 5 to 100° with a step size of 0.03°.

Thermo-diffraction patterns from 25 to 400 °C (298 to 673 K) were performed with a temperature camera Anton Paar HTK2000 with a platinum strip heater. For the low-temperature measurement (14–300 K), the camera stage was Oxford Phenix cryostat. For thermo-diffraction patterns, the samples were fixed with ethanol at a Cu holder and vacuum atmospheres. The collected data range was from 5 to 80° with 0.0334 steps and Bragg–Brentano focusing. These diffractometer and thermo-diffractometer measurements were operated at the X-ray Diffraction Center, Complutense University of Madrid, Spain. The data were characterized and refined using Rietveld analysis computer programs Crystal Impact MATCHi program^39^ and FullProf package suite.^40^ The geometrical crystal structure models were created using VESTA (visualization for electronic and structural analysis).^41^

SAED measurements were carried out in a JEOL JEM-2100 transmission electron microscope operating at 200 kV. The composition analysis of the samples was performed by XEDS in the same microscope using the Oxford Inca microanalysis system. High-resolution TEM (HRTEM) images were obtained in a JEOL JEM-3000F transmission electron microscope with an acceleration voltage of 300 kV and structural resolution of 1.7 Å. Image simulation was accomplished with the TempasX software package (Total Resolution LLC) using a multislice calculation method to simulate the high-resolution images obtained in the JEM-3000F at different values of thickness and defocus.

### 4.4 Magnetic Characterization

The magnetic characterization has been performed in a Quantum Design SQUID magnetometer. Hysteresis cycles have been measured at 2 at 5 T and 14 T. The thermal dependence of the magnetic susceptibility at 500 Oe has been measured from 2 to 300 K. Previously, for each measurement, a 3 T demagnetizing field was applied. The characterizations were performed at the Instituto de Magnetismo Aplicado, Complutense University of Madrid, Spain, and in the Servicio General de Apoyo a la Investigación-SAI, Universidad de Zaragoza, Spain.

## Supporting Information Available

Refined crystal structure for all the samples, Cr–Cr distances, thermogravimetric results, thermal dependence of the cell parameters, original and simulated HRTEM images, and hysteresis cycles of the CC1 and CC3 samples. (PDF)
