# Supplementary material for: Coexistence of Two Spin Frustration Pathways in the Quantum Spin Liquid Ca10Cr7O28
Source: Inorg Chem. 2022 Oct 3;61(41):16228–38. doi: 10.1021/acs.inorgchem.2c01831 (PMC9580002; doi:10.1021/acs.inorgchem.2c01831)
Supplement: Supplementary file 1 — ic2c01831_si_001.pdf [file ic2c01831_si_001.pdf]

Coexistence of two spin frustration pathways in the quantum spin liquid $Ca_{10}Cr_7O_{28}$

Dhoha R. Alshalawi,[1,3] José M. Alonso,[1,2] Angel R. Landa-Cánovas,[2] Patricia de la Presa.[1,3]*

1 Institute of Applied Magnetism, UCM-ADFI-CSIC, A6 22,500 km, 28230 Las Rozas, Spain

2 Institute of Material Science of Madrid, CSIC, 28049 Madrid, Spain

3 Department of Materials Physics, Complutense University of Madrid, 28040 Madrid, Spain

Corresponding author e-mail: pmpresa@ucm.es

S1. XRD structural characterization

a) Rietveld refinements: The table S1 shows the results of the Rietveld refinements at room temperature performed in the four samples: CC3 (pure sample), and CC1, CC2, and CC4. The atomic positions, isotropic thermal displacement B values and occupation numbers as well as Bragg R-factor, weighted Chi2 and fractions of impurity phases are shown in the tables.

Table S1: Tables of Refined Crystal Structure of other $Ca_{10}Cr_7O_{28}$ Samples (CC3(2.85), CC1 (3:1), CC2(2.9:1), and CC4(2.8:1)). The Crystal system and Space group: Trigonal (hexagonal axes), R3c (161), at 300K. The numbers in parenthesis are the standard deviations.

Sample CC3 (2.85:1)

Bragg R-factor	Weighted Chi2	Fract(%) of one Phase
14.5	4.53	100.00(0.00)

Atoms	X	Y	Z	B	Occ.
Ca1	0.2907(5)	0.1635(6)	-0.05531(13)	0.015(2)	1
Ca2	0.1918(6)	-0.1812(6)	0.00496(13)	0.00326	1
Ca3	0.3975(4)	0.1907(7)	0.03681(12)	0.00279	1
Ca4	0.66670	0.33330	0.1062(3)	0.012(2)	1
Cr1	0.3137(3)	0.1461(4)	0.13810(10)	0.00441	1
Cr2	0.1813(5)	-0.1350(4)	-0.09459(12)	0.0204(13)	1
Cr3	0.00000	0.00000	-0.0020(5)	0.049(10)	0.31040
Cr4	0.00000	0.00000	0.0332(2)	0.00201	0.681(6)
O1	0.00000	0.00000	-0.0467(13)	0.00702	0.35972
O2	0.00000	0.00000	0.0806(8)	0.00534	0.63215
O3	0.268(2)	0.097(3)	0.0921(6)	0.113(16)	1
O4	0.2664(14)	0.2631(13)	0.1561(4)	0.00806	1
O5	0.2649(14)	-0.0072(13)	0.1610(3)	0.00547	1
O6	0.4887(10)	0.232(2)	0.1499(3)	0.015(5)	1
O7	0.1401(17)	-0.1303(16)	-0.0446(3)	0.00130	1
O8	0.2396(16)	-0.2436(17)	-0.1041(3)	0.01251	1
O9	0.3076(13)	0.0422(13)	-0.1048(4)	0.00446	1
O10	-0.0202(16)	-0.2060(14)	-0.1102(5)	0.051(7)	1
O11	0.0343(16)	0.1575(11)	0.0222(4)	0.00396	1

Sample CC1 (3:1)

Bragg R-factor	Weigthed Chi2	Fract(%) of Phases		
		$Ca_{10}Cr_7O_{28}$	$CaCrO_4$	CaO
5.36	3.63	99.52	0.28	0.21

Atoms	X	Y	Z	B	Occ.
Ca1	0.2823(5)	0.1536(7)	-0.06051(15)	0.030(3)	1
Ca2	0.1975(6)	-0.1932(6)	0.00094(14)	0.014(2)	1
Ca3	0.3872(5)	0.1830(7)	0.03406(13)	0.011(2)	1
Ca4	0.66670	0.33330	0.1013(4)	0.011(3)	1
Cr1	0.3108(3)	0.1396(5)	0.13297(12)	0.0192(18)	1
Cr2	0.1777(5)	-0.1373(4)	-0.09900(11)	0.0097(15)	1
Cr3	0.00000	0.00000	0.0037(2)	0.02329	0.74105
Cr4	0.00000	0.00000	0.02680	0.02775	0.24443
O1	0.00000	0.00000	-0.0428(10)	0.00676(2)	0.73920(4)
O2	0.00000	0.00000	0.0738(19)	0.01618	0.24434
O3	0.2750(14)	0.0944(15)	0.0881(4)	0.023(6)	1
O4	0.2274(14)	0.2280(14)	0.1452(4)	0.00506	1
O5	0.2761(16)	-0.0156(14)	0.1539(4)	0.01145	1
O6	0.4902(11)	0.2439(17)	0.1418(3)	0.00230	1
O7	0.1585(18)	-0.1203(17)	-0.0561(3)	0.017(7)	1
O8	0.2501(18)	-0.2417(18)	-0.1110(4)	0.010(6)	1
O9	0.3022(14)	0.0351(14)	-0.1127(5)	0.00229	1
O10	0.0123(15)	-0.2200(14)	-0.1181(4)	0.005(7)	1
O11	0.007(3)	0.1506(16)	0.0178(7)	0.019(7)	1

Sample CC2 (2.9:1)

Bragg R-factor	Weigthed Chi2	Fract(%) of Phases		
		$Ca_{10}Cr_7O_{28}$	$CaCrO_4$	CaO
14.9	3.98	99.77	0.15	0.09

Atoms	X	Y	Z	B	Occ.
**Ca1**	0.2802(7)	0.1536(11)	-0.0618(2)	0.021(4)	1
**Ca2**	0.1962(10)	-0.1935(10)	-0.0000(2)	0.00507	1
**Ca3**	0.3842(7)	0.1855(11)	0.0343(2)	0.005(2)	1
**Ca4**	0.66670	0.33330	0.1019(6)	0.016(4)	1
**Cr1**	0.3147(5)	0.1438(7)	0.13191(17)	0.002(2)	1
**Cr2**	0.1803(7)	-0.1344(6)	-0.09853(18)	0.00409	1
**Cr3**	0.00000	0.00000	0.0096(7)	0.08316	0.59646
**Cr4**	0.00000	0.00000	0.0354(7)	0.01775	0.39444
**O1**	0.00000	0.00000	-0.0746(14)	0.00988	0.64007
**O2**	0.00000	0.00000	0.067(3)	0.00536	0.35742
**O3**	0.239(2)	0.079(3)	0.0861(7)	0.080(18)	1
**O4**	0.236(2)	0.239(2)	0.1441(6)	0.00823	1
**O5**	0.261(3)	-0.029(2)	0.1523(6)	0.00011	1
**O6**	0.4940(16)	0.246(4)	0.1429(5)	0.00990	1
**O7**	0.158(3)	-0.134(2)	-0.0548(5)	0.00758	1
**O8**	0.253(3)	-0.242(3)	-0.1174(6)	0.00476	1
**O9**	0.286(2)	-0.003(2)	-0.1022(7)	0.020(10)	1
**O10**	0.010(2)	-0.220(2)	-0.1146(7)	0.00846	1
**O11**	0.024(4)	0.165(2)	0.0104(8)	0.088(14)	1

Sample CC4(2.8:1)

Bragg R-factor	Weigthed Chi2	Fract(%) of Phases	
		$Ca_{10}Cr_7O_{28}$	$CaCrO_4$
19.2	4.84	98.78	1.22

Atoms	X	Y	Z	B	Occ.
**Ca1**	0.2834(11)	0.1521(13)	-0.0614(3)	0.029(5)	1
**Ca2**	0.2182(12)	-0.1829(13)	0.0025(3)	0.018(4)	1
**Ca3**	0.3696(10)	0.1918(14)	0.0353(3)	0.002(3)	1
**Ca4**	0.66670	0.33330	0.1037(7)	0.026(7)	1
**Cr1**	0.3110(7)	0.1543(12)	0.1339(2)	0.009(3)	1
**Cr2**	0.1843(10)	-0.1390(9)	-0.0985(2)	0.003(3)	1
**Cr3**	0.00000	0.00000	0.0177(10)	0.05444(10)	0.69039
**Cr4**	0.00000	0.00000	0.0719(13)	0.01583	0.28816
**O1**	0.00000	0.00000	-0.0132(18)	0.02266(4)	0.66247(7)
**O2**	0.00000	0.00000	0.124(4)	0.00544	0.28771
**O3**	0.304(5)	0.055(5)	0.0846(12)	0.02(3)	0.65(6)
**O4**	0.230(3)	0.213(3)	0.1443(8)	0.00028	1
**O5**	0.285(4)	-0.022(3)	0.1487(10)	0.036(13)	1
**O6**	0.479(3)	0.227(4)	0.1351(8)	0.00125	1
**O7**	0.140(4)	-0.117(4)	-0.0488(8)	0.00064	1
**O8**	0.258(4)	-0.245(3)	-0.1136(9)	0.00716	1
**O9**	0.275(3)	-0.011(3)	-0.1044(9)	0.001(17)	1
**O10**	0.081(3)	-0.175(3)	-0.0959(8)	0.010(18)	1
**O11**	0.038(3)	0.173(3)	0.0132(9)	0.003(11)	1

b) The Cr-Cr distances: Table S2 shows the Cr-Cr distances for all the samples. These distances are calculated from the data obtained in Table S1. The table illustrates that the shortest distances between Cr1-Cr2 are in the direction of the ladder and not in the Kagome planes.

Table S2: Cr-Cr Distances (Å) of Samples. The numbers in parenthesis are the standard deviations.

	Samples Cr-Cr Distances (Å)			
**Cr bonds**	**CC3**	**CC1**	**CC2**	**CC4**
**Cr1-Cr1**	5.07175	5.02948(4)	5.08950(4)	5.02531(7)
**Cr1-Cr1**	5.70427(2)	5.75640(3)	5.68977(5)	5.74878(8)
**Cr2-Cr2**	5.12764	5.10273(4)	5.10057(5)	5.24259(8)
**Cr2-Cr2**	5.67550(3)	5.69328(4)	5.70089(4)	5.56461(8)
**Cr3-Cr4**	1.33944	0.88095	0.98204	2.06510(6)

S2. Calorimetric results

Thermogravimetric results of the sample $Ca_{10}Cr_7O_{28}$ in reducing atmospherer H/He (0.2/0.3 atm.) from room temperature up to 900 °C. The final products were analysed by XRD was always the same mixture of CaO and Cr2O3 oxides. This result shows that the material is very stable up to 400 ºC. The oxygen content was determined from the weight difference between the starting material and the final products.

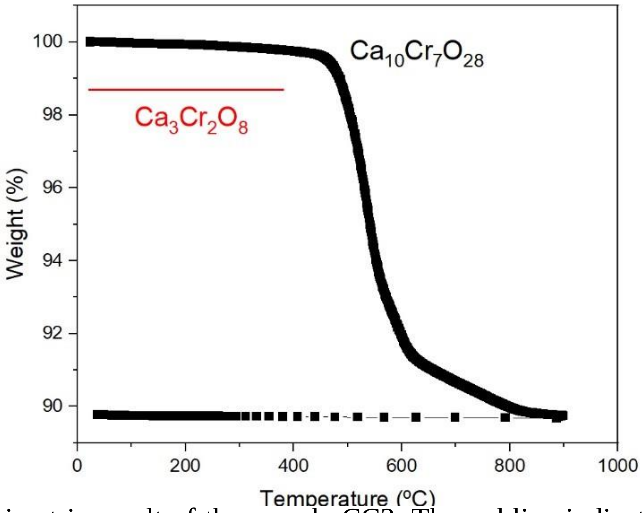

Figure S1. Thermogravimetric result of the sample CC3. The red line indicates the mass percent of $Ca_3Cr_2O_8$ and the black line of $Ca_{10}Cr_7O_{28}$.

S3. XRD thermo-diffraction

The following figures shows the crystal cell parameters a, c and the cell volume calculated from the Rietveld refinements of the XRD spectra measured from 15 to 673 K.

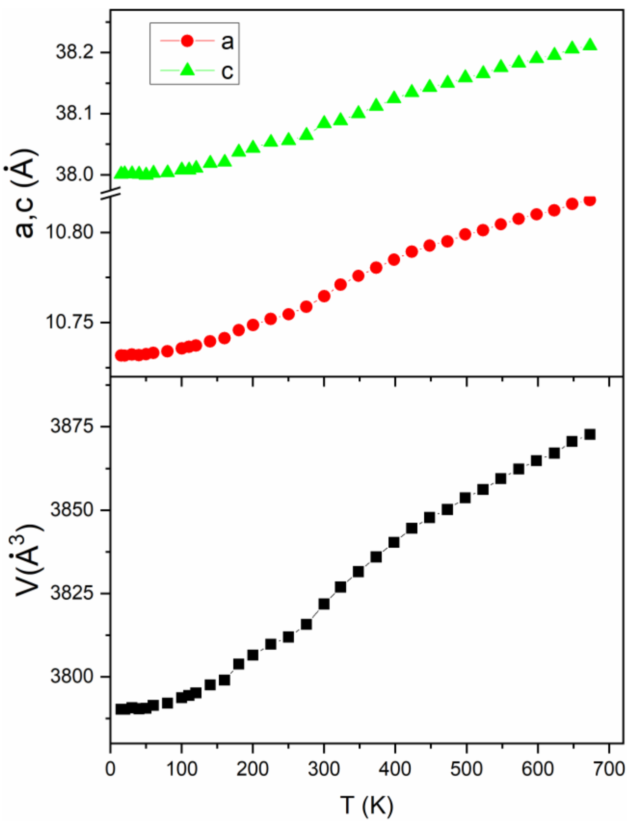

Figure S2. Thermal dependence of the cell parameters a, b, and c (top) and the cell volume (bottom) obtained by Rietveld of thermo- diffractions at temperatures between 15 and 673 K.

S4. High Resolution Microscopy (HRTEM)

a) HRTEM image: The following figure is the original image along [100] direction and the corresponding Fast Fourier Transform (FFT) of the sample CC3. As can be seen, there is radiation damage of the crystals and, consequently, an important noise contribution Therefore, image-processing techniques have been used to eliminate noise. They consist in obtaining the fast Fourier transform of the image (FFT) and filtering out most of the noise using digital masks to reconstruct the image from just the structural maxima by an inverse fast Fourier transform (IFFT). The elimination of the noise of this image resulted in the Figure 9 of the main manuscript.

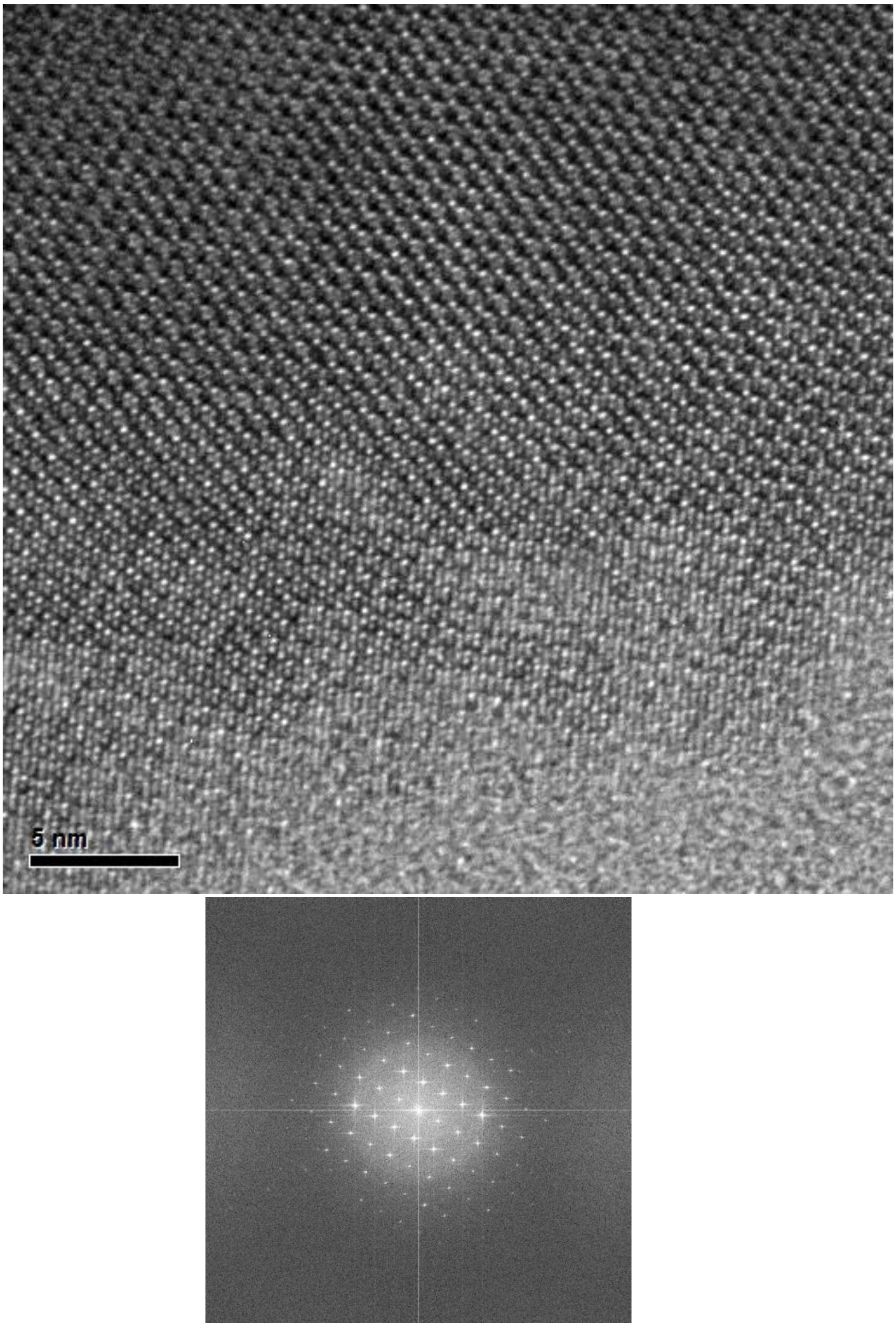

Figure S3. Original HRTEM image oriented along the [100] zone axis without noise reduction corresponding to the processed image of Fig. 9 of the paper. Notice the crystal damage at left down corner. Under it is its corresponding FFT (Fast Fourier transform).

b) HRTEM simulate images: In the inset of Figure 9 of the manuscript**,** there is an image simulation for a crystal generated with the atomic parameters from the Rietveld refinement data, with 125 Å thickness at -600 Å defocus, calculated by multislice methods to take account of the dynamical effects of electron scattering.

For that purpose, a whole series of images have been calculated with the parameters of the TEM microscope and varying both thickness and defocus, the two unknown variables. The results are exposed in Figure S4, where the images have been calculated for four thicknesses ranging from 50 to 125 Å and the objective lens defocus from -100 Å to -800 Å. The calculated image that fits better to the experimental image of Figure 9 is the one corresponding to a thickness of 125 Å and an objective lens defocus of -600 Å.

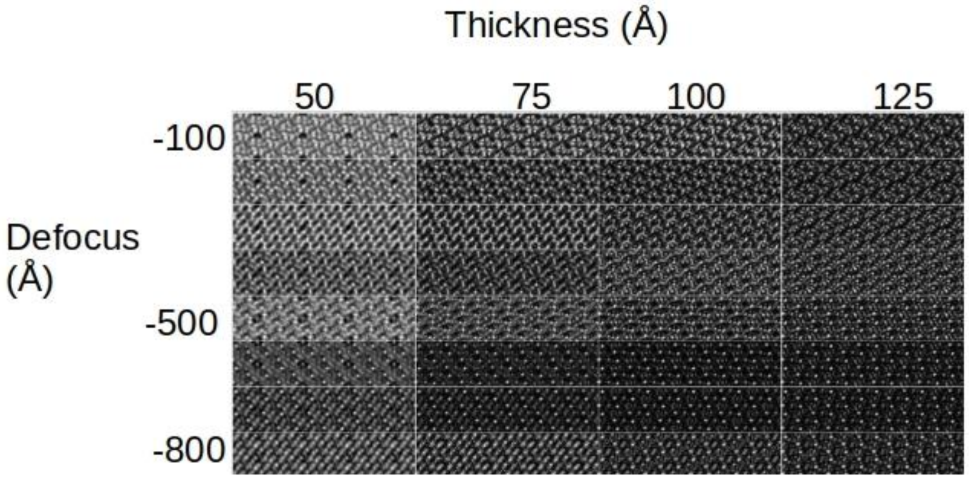

Figure S4: HRTEM simulated images for thickness 50-125 Å and defocus -100 - 800 Å

S5. Magnetic properties

The figure S5 shows the hysteresis curves at 5 K of samples CC1 and CC3. As can be seen, sample CC1 (3:1) has 19.1 emu/g at 5 T, 12% smaller than the magnetization of CC3 (2.85:1), 21.7 emu/g. The decrease in the magnetization of CC1 cannot be explained by normalizing with the mass of the segregated phase since there is only 1.5% of CaO excess. Therefore, it suggests that the CaO excess plays a major role in the magnetic interaction of the Cr-Cr cations.

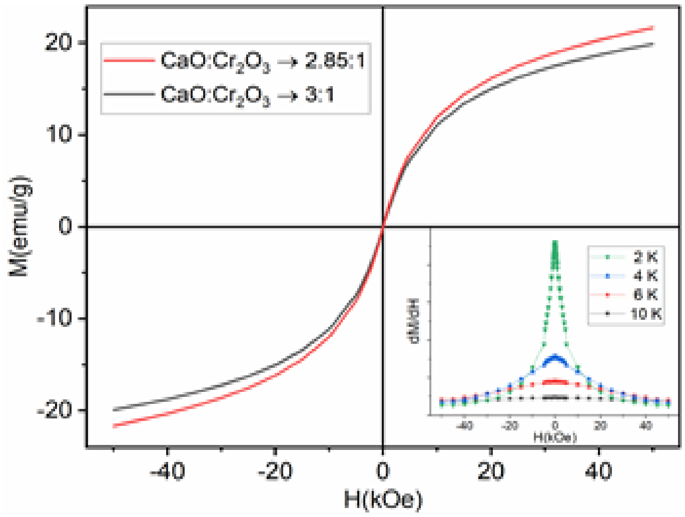

Figure S5: Hysteresis loops at 2 K and 5 T of the stoichiometric (CC3, red line) and non-stoichiometric (CC1, black line) samples. The inset shows the susceptibility as a function of the field for different temperatures of the stoichiometric samples.
